# Priority populations’ experiences of the accessibility and inclusion of recreation centres: a qualitative study

**DOI:** 10.1186/s12889-023-17595-3

**Published:** 2024-01-17

**Authors:** Elise Rivera, Cynthia Smith, Kylie D Hesketh

**Affiliations:** Institute for Physical Activity and Nutrition (IPAN), School of Exercise and Nutrition Sciences (SENS), Deakin University, Locked Bag 20001, Geelong, VIC 3220 Australia

**Keywords:** Physical activity, Leisure centre, Fitness centre, Adults, Disadvantaged populations, Active recreation, Underserved populations

## Abstract

**Background:**

Although the health benefits of physical activity are well documented, certain priority populations are often disproportionately insufficiently active and at higher risk of poor health. Recreation centres have the potential to provide accessible and supportive environments for physical activity for all. However, little is known about priority populations’ experiences of these venues and their views of how accessibility and inclusion can be optimised. This study aimed to gain in-depth insights of recreation centre experiences and potential strategies for improving inclusion and accessibility amongst priority populations (women, older adults, ethnic minorities, persons living with disabilities/additional needs, individuals identifying as LGBTQIA+, low socio-economic position).

**Methods:**

This qualitative study (2021–2022) involved 18 semi-structured individual interviews with adult priority population users of recreation centres (50% 65 + years, 61.2% female) in one Melbourne municipality. Participants were asked to discuss their positive and negative experiences at the centres and to identify strategies for enhancing accessibility and inclusion. Interviews were audio-recorded and transcribed verbatim. Content analysis was performed for data analysis.

**Results:**

While many participants had positive views of the facilities and programs at the centres, as they met their needs, they also had suggestions for improving accessibility and inclusion. Similarly, most participants were happy with the communications, felt included, and perceived the culture positively. Those who did not feel included at the centres offered many potential strategies for changing the culture, modifying communications (e.g., signage), and establishing partnerships for better access and inclusion.

**Conclusions:**

The present study adds to essential knowledge concerning priority populations’ experiences of recreation centres. For recreation facilities that were generally perceived as having positive inclusion and accessibility, the findings nonetheless highlighted suggestions for further enhancement. These strategies may be useful more broadly for improving accessibility and inclusion, thereby promoting physical activity and ultimately health for all.

**Supplementary Information:**

The online version contains supplementary material available at 10.1186/s12889-023-17595-3.

## Background

The numerous health and wellbeing benefits of physical activity are well documented [1]. However, according to the World Health Organization, 28% of adults fail to achieve physical activity guidelines [2]. Priority populations have been defined as various groups across society, who experience social disadvantage and inequalities due to health inequity (e.g., avoidable, unfair differences in health status) [3]. Government and non-government organisations may provide targeted services, policies and programs to equalise opportunities and promote the health of equity-deserving communities [4]. Certain priority populations, such as individuals experiencing low socio-economic position (SEP) [5], culturally and linguistically diverse groups [6], persons living with disabilities [7, 8], women [9], persons identifying as LGBTQIA+ [10], and older adults [11] are typically less likely to be sufficiently active and are at increased risk of mortality and morbidity in comparison to their counterparts.

Many factors can influence physical activity engagement [12]. For example, research shows that having access to exercise facilities, local opportunities for physical activity (e.g., community-based exercise classes, local recreation centres), and social support can encourage adults to be physically active [13–18]. The literature has indicated that access to supportive recreation facilities and programs care critical for enabling individuals to implement health behaviours [15]. Community settings, such as recreation centres (e.g., leisure and aquatic centres, gyms), may be particularly relevant for providing physical activity opportunities among diverse populations given their proximity and diverse offerings (e.g., organised and non-organised activities that can be performed individually or in groups) [19, 20]. Additionally, the social environment provided by recreation centres may foster social connections and provide further behavioural and social reinforcement for physical activity [17].

Providing accessible and supportive environments for physical activity amongst all population groups is a major global health priority [21, 22], and recreation centres can play a key role in this. Memberships at recreation centres have risen globally in recent years [23, 24]; however, these settings remain underutilised by certain priority populations, such as persons living with disabilities [25–27], people identifying as LGBTQIA+ [28], and older adults [29]. While we know these priority populations face additional barriers to physical activity [7, 30–35] and have been shown to be less likely to use these centres due to challenges concerning accessibility and inclusion [27, 36–38], there is a paucity of research exploring their experiences and barriers to use. A recent scoping review investigated facilitators and barriers to exercise in fitness centres among adults living with and without disabilities [39]. Findings showed that facilitators for people living with a disability included accessible design, specially trained staff, assistance from instructors, tailored exercise programs, opportunities to socialise, specialised equipment, and an inclusive environment [39]. Conversely, barriers among this sub-population included poor transport options, poor accessibility in or around facilities, high cost, lack of knowledge about accessible facilities available, lack of skilled instructors, management not being actively inclusive, lack of tailored classes/adaptive programs, stigma, unsuitable fitness equipment, and lack of social support [39]. Studies have also found that stigma or marginalisation, a lack of accessible facilities, and limited inclusion were barriers to recreation centre use among CALD groups [33], women [34, 35], and people identifying as LGBTQIA+ [32].

To our knowledge, no studies have investigated the collective recreation centre experiences of multiple priority populations who may be more likely to experience accessibility and/or inclusion barriers. Further, the aforementioned studies primarily focused on factors that encourage or discourage exercise at fitness centres and did not investigate participants’ views and perceptions of potential strategies for improving accessibility, inclusion, and service delivery in these settings. It is critical for the voices and needs of priority populations to be reflected in the (re)design of recreation centres and the provision of recreation services to reduce inequalities in physical activity opportunities and use of recreation centres, as well as to address accessibility and inclusion barriers in these settings. Therefore, this qualitative study sought to gain in-depth insights from priority populations regarding their experiences of recreation centres and potential strategies for improving inclusion and accessibility.

## Methods

This research was conducted as part of a broader evaluation of recreation centres located in one local government area (LGA; population ~ 175,000) in Melbourne, Australia. This evaluation originated in response to the Auditor-General’s report on Local Government Service Delivery: Recreational Facilities (2016) [40], which called for the evaluation of recreation centres to shift from focusing on evaluating outputs (e.g., attendance) to evaluating outcomes that help councils better understand whether centres are meeting the service needs of the community and attaining councils’ broader social, health and wellbeing goals. This evaluation was initiated by the LGA and the service provider of the recreation centres in the LGA, with academic collaborators brought in to examine the impact of the recreation centres on the health and wellbeing of the LGA’s community. Data were collected between November 2021 and February 2022. This paper adheres to the Consolidated Criteria for Reporting Qualitative Research (COREQ) checklist [41]. Compensation was not provided for interview participation.

### Setting and participants

Participants were users (either casual or regular) of at least one of three recreation centres included in this study but were not necessarily members of the centres or residents of the LGA where the centres were located. The three recreation centres were funded by the LGA. While some facilities slightly varied across the centres, they generally included: a fully equipped gym; a functional training space; group fitness studios; a café; pool(s); sauna; and stadium(s) for various sports. There were a range of group fitness and wellbeing classes and personal training services offered at all three centres.

Using purposive convenience sampling, a targeted sample of facility users from specific priority population groups (women, older adults, persons identifying as LGBTQIA+, living with disability/additional needs, identifying as Aboriginal and/or Torres Strait Islander, experiencing low SEP, and/or of a CALD background), who may face accessibility and inclusion challenges were recruited in October 2021. The researchers provided a study invitation, Plain Language Statement with study information, and a link to an online consent form to the centres’ recreation service provider to email to relevant organisations that utilise their facilities, for dissemination to their members. Recruitment materials (e.g., flyers) were developed by the recreation service providers and provided to the following targeted organisations. There were three state-based organisations that service people with disabilities. There was a local organisation that services low SEP populations, persons identifying as LGBTQIA+, CALD individuals, and young adults. Other targeted organisations included: a group for adults aged 50 + years established at the recreation centres; a local all-women’s group; and a city-based organisation that services people from CALD and low SEP backgrounds. The research team contacted those who completed the online consent form by phone or email to arrange an interview time and date. Due to high engagement from the adults aged 50 + years group, participants were capped at six to ensure the needs of that population group were not over-represented in the data, and purposeful sampling ensured an even gender distribution. Thirty-four people from the different priority populations provided written informed consent and were contacted; however, only one participant identifying as LGBTQIA + and one person with a CALD background consented, so there was not a balanced distribution of potential participants from each priority population group, although many persons identified with more than one priority population group. The recreation service provider prompted target organisations to address the underrepresentation of certain priority populations, but this was unsuccessful for recruiting participants from the non-represented and under-represented population groups. The research team, which consisted of three people, did not impact recruitment as it was done through the recreation service provider.

Fourteen people were unreachable after two attempts. Interviews were scheduled for 20 people, of which two were unreachable at the time of the interview and did not respond when contacted to reschedule. The final sample consisted of 18 participants (described in Results).

### Data collection

Before starting, participants were reminded that the interview was confidential, given an overview of the interview procedure and encouraged to speak freely as they were considered the “expert”. ER, a Research Fellow trained in qualitative data collection, conducted all interviews and did not have any conflicts of interest, biases, or existing relationships with participants. Interviews were conducted in English via Zoom or Skype for Business (depending on participants’ preferences), audio-recorded, and transcribed verbatim. Interview length ranged between 17 and 52 min (average 37 min).

An interview schedule (see Additional file 1) was developed by the research team with questions adapted from the *7 Pillars of Inclusion*, a model created by Play by the Rules and Sport Australia to guide Australian sports clubs and associations with addressing diversity and inclusion to create inclusive sports as a means to foster inclusive communities [42]. The model contains the following pillars: choice; partnerships; communications; policies; opportunities; access; and attitude [42]. The adapted model used in the present study was designed to suit the recreation setting and included questions across the following pillars: access and choice (focused on facilities); access and choice (focused on programs); communications; attitude; and partnerships/opportunities. Principles of semi-structured interviews were applied [43]. The interview schedule was piloted with a male adult (18–34-year age group) to ensure acceptability and comprehension of the questions and to gain insights regarding the structure of the interviews and approximate duration. No further changes were made to the interview schedule based on the pilot interview. For the main set of interviews, field notes were made after each interview by the interviewer. Participants were not provided with their transcripts.

### Data analysis

Transcriptions were entered and coded in NVivo 20 (QSR International Pty Ltd). Given the descriptive aims of this study, data were analysed deductively using content analysis [44, 45]. A preliminary coding framework was developed by two researchers (ER, CS), based on the interview questions (e.g., Are there any barriers you have faced when using the facilities?), which included potential responses (e.g., inaccessible equipment, lack of assistance with equipment use) according to existing research [39]. Throughout the coding process, the framework was iteratively adapted as new content emerged. After the transcripts were coded, they were grouped into (sub)categories. CS, ER, and KDH discussed the codes, categories, and interpretation of the data. The findings were repeatedly checked against the transcripts to validate the analyses, particularly at the final analysis stage. CS and ER identified illustrative quotes and discussed them with KDH to identify those that best reflected the themes.

## Results

As shown in Table 1, the 18 recruited participants represented women (n = 11), older adults (n = 9), individuals living with a disability or additional needs (n = 8), low SEP (n = 7; identified by having a low-income Healthcare/Pension card), CALD (n = 1), and LGBTQIA+ (n = 1). No participants identified themselves as Aboriginal and/or Torres Strait Islander. It should be noted that many individuals represented multiple priority population groups.

**Fig. 1:**
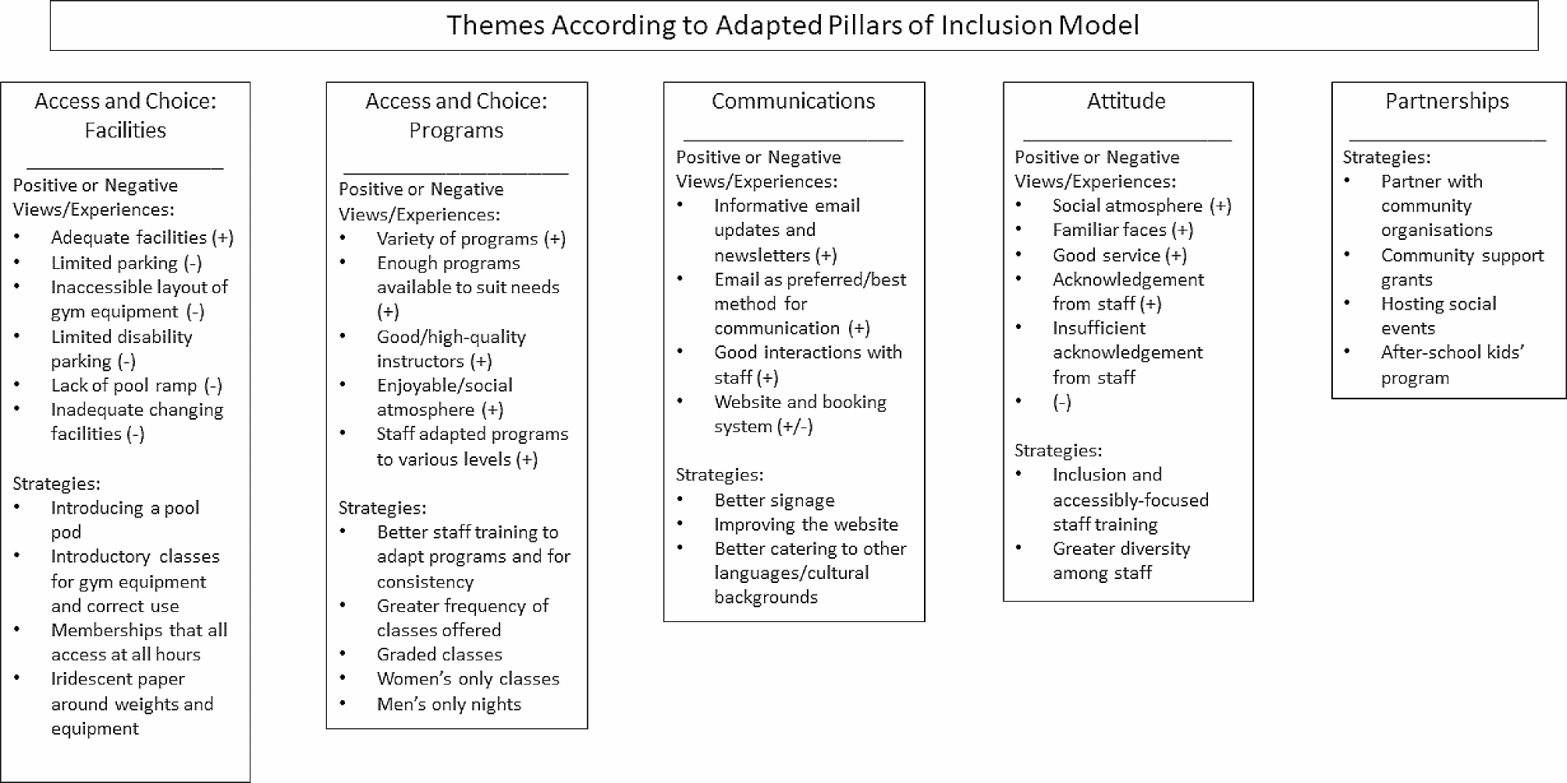
Themes according to adapted pillars of inclusion model

Findings below are presented for each of the categories investigated under the adapted *Pillars of Inclusion* model utilised in this study (Fig. 1). In the subsequent sections, illustrative quotes are provided. Given that most participants identified across multiple priority populations, these details are not included with the quotes to avoid potential identifiability.

**Table 1:** Demographic characteristics of participants (n = 18)

Age group	n (%)
18–34 years	2 (11.1%)
34–64 years	7 (38.8%)
65 + years	9 (50.0%)
Gender	
Male	7 (38.8%)
Female	11 (61.2%)
Aboriginal and/or Torres Strait Islander	0 (0.0%)
Language other than English spoken at home^a^	1 (5.3%)
LGBTQIA+	1 (5.3%)
Reports disability or additional needs	8 (44.4%)
Has a pension or Healthcare Card^b^	7 (38.8%)
Member or user of the centre(s)^c^	
Member	11 (61.1%)
User	7 (38.8%)

^a^ One participant indicated Urdu and English as the main languages spoken at home

^b^ Indicates low income

^c^ Indicates that participants hold a membership at the recreation centre(s) in the LGA

### Access and choice: facilities

Participants generally rated recreation centres based on whether their needs were met by the variety of facilities and types of equipment available. Good upkeep of the equipment was also commonly cited by participants, notably those who were older (65 + years) and without a disability or additional needs, as reasons why the facilities were appealing.

It offers so many facilities. It’s got swimming. It’s got gym. It’s got an exercise physiologist. It’s got group classes.

The pools are very, very clean. It’s not dirty. It’s well-maintained.

However, there were accessibility barriers. Half of participants specified limited parking or limited disability parking as a barrier for accessing the recreation facilities.

The parking is absolutely horrible. When it’s busy, if you’ve got a wheelchair, and you’ve got to get outside of a car and the disability spots are taken, there’s just nowhere where you can park.

Some participants mentioned difficulties with manoeuvring around the equipment and centres due to inaccessible layout of equipment and inadequate accessible facilities (e.g., no disabled changing rooms, no pool ramp). They had to rely on staff for assistance or facilities were self-manageable but unappealing, detracting from the recreation experience. These barriers were predominately mentioned by those living with a disability and who were 65 + years.

All the treadmills and the cross trainers, it was just a line of them side-by-side. Say there were ten side-by-side, for a disabled person, the only way you could get onto it was one on each line and that was the end one because they were tightly side-by-side and up against the wall.

Individual participants who identified as living with a disability or as an older adult, provided some recommendations for increasing recreation facilities’ accessibility, which included: offering classes to acquaint people with the gym equipment and correct use; ensuring all membership categories allow access to facilities at all opening hours; adding iridescent paper around weights for better visibility; and introducing a Pool Pod, a device to assist pool entry independently without need for staff assistance.

### Access and choice: programs

Most participants spoke about having a variety of classes available to meet their various needs and ease of participation as being important for creating positive experiences with the programs. Having enough programs suited to them, good instructors, and an enjoyable social environment were further reasons why participants perceived programs as appealing and accessible.

There’s enough classes that you can pick and choose what you want both in terms of the programs within the class and finding the group leaders who teach well.

Half of participants noted that staff adapted the programs to cater to their level and abilities. However, when describing ways of improving the accessibility and inclusion of existing programs, several participants across all priority groups, especially persons living with a disability and adults aged 65 + years, felt that instructors could be given better training and support to tailor programs to suit their needs and create better consistency across the classes.

There are a few instructors there which I’ve had in the past where they generally don’t consider older members. You know members’ health or physical problems, whatever it is; they assume you should be able to do it.

… there doesn’t seem to be any checking on what the instructors do. Each instructor is very different to the other one; there doesn’t seem to be a program or whatever that they follow.

Some participants with a disability or who were 65 + years also mentioned that they would like some specific programs to run more often (e.g., wheelchair football, classes for people with vision impairment), particularly when popular classes became full. Further, there were targeted suggestions by single participants for additional programs to improve accessibility and inclusion, which included graded classes, women’s only classes, and men’s only nights.

### Communication

When asked about communications they receive from their recreation centre, most participants stated that they found the monthly email updates clear and informative and considered email as the preferred and more accessible method of communication.

Some organisations bombard you with emails, which is very irritating. But they [this recreation centre] would always send a message that was very informative and very clear.

Email is the best or is the easiest, or most accessible I think.

Several participants also mentioned that they had good experiences overall when communicating with the staff, noting that they were approachable and helpful.

I don’t always see the same people all the time, but when you’re checking in and checking out, they’re all very friendly and cheerful and very helpful.

Several participants mentioned the importance of websites being kept up to date and being easy to navigate and user-friendly, particularly in regard to centre/class timetables, which was frequently accessed information.

The website I don’t find user friendly at all, I get quite overwhelmed with the website… I’m always struggling with the timetable, what’s the new timetable of the classes, because apparently it had updated but they didn’t have the updated brochures, and the brochures that they had were different to the timetables that were online.

Several participants provided suggestions for how communications could be enhanced to increase accessibility and inclusion, particularly through signage, improving the website (e.g., adding information about accessible facilities at the centres), and presenting information in multiple languages.

I mean even as you enter the change room, the F for female and M for male, neither of my parents would be able to see them – even though they’re probably a meter tall – because they’re the same colour as the background wall. So, just little things like that make a big difference in accessibility. Yellow lines painted on the floor, guiding them to the entry to the pool or to the gym. Maybe red to the gym and yellow to the pool.

I know some providers that run facilities do have information on their websites in regard to accessible toilets, accessible car parking. Sometimes even more accessible times where it’s not so busy – off peak, and so on where people with disabilities who are a little bit more, I suppose, self-conscious or aware, might be more comfortable in going to these facilities for a swim or to do a workout.

you do have a need to have signage in multilingual where possible, particularly in areas that are high traffic...

### Attitude

Centre culture and environment were considered important influences on how included and comfortable participants felt at the recreation centres. Some participants, especially older adults, stated that it was the social atmosphere and sense of familiarity from seeing other returning users that made them feel included. Having good service and acknowledgement from staff and friendly instructors were also stated as important factors for making participants feel comfortable at recreation centres.

People who go there are going there for the same reason as you; they’re going there for social interaction. They’re going there for their mind. They’re going there for their body. And so therefore we’re all there for a common purpose. And the instructors who attend these are of like mind. So it’s a very, very comfortable experience. I don’t feel left out.

I’m always acknowledged when I go in and that’s important just to be made feel like a human being. Because when you can’t move and that, you do get a bit down. And it’s just nice sometimes when someone on the staff says, ‘Hello, how are you?’ And they don’t realise how important that is.

When asked to describe ways that participants could feel more comfortable and included and to make the culture more inclusive, many participants, particularly those living with a disability, suggested better staff training and inductions around inclusion and accessibility.

There’s quite a bit of turnover of staff at the gym and I think it would be good if people, maybe as part of their induction, did some training about interacting with people with disabilities. I think that would keep it consistent and people would have hopefully more idea about how to help and assist people.

Some participants noted that the culture of the centres could be improved by having a greater diversity across staff that reflected the priority populations.

When I walk into a facility, as a person with disability, if I can see a person with disability behind the desk as a staff member, if I can find someone who is from a cultural background that looks just like me, and it can reflect me, I can hold a mirror to the workforce that reflects the community.

### Partnerships/opportunities

When asked about partnerships and opportunities that could enhance accessibility and inclusion, many participants discussed partnering with a range of community organisations, including those that service priority populations.

…the more relationships they can have with community organisations the more inclusive the organisation will be and the more comfortable people with specific needs will feel to go there.

Additionally, there were some other targeted suggestions by individual participants in certain priority populations (notably low SEP, older adults) for recreation centres to be a hub to provide practical support beyond physical activity and recreation to priority populations, (e.g., social worker/community development officer) and social support (e.g., a gathering place for social support/social networks).

## Discussion

This study provided an in-depth understanding of priority populations’ experiences of recreation centres and their suggestions for improving the accessibility and inclusion of these settings. Of the *Pillars of Inclusion*, ‘access and choice’ (for both facilities and programs) and ‘attitude’ appeared to be most important for the different priority populations as participants most commonly spoke of these pillars when discussing their experiences at the recreation centres and recommendations for improving accessibility and inclusion to meet their needs. Although the key themes were generally similar across the various priority populations included in this study, there were some nuances concerning the nature of the accessibility and inclusion barriers and suggestions for improved service delivery. For example, across the pillars, persons living with a disability and additional needs discussed more physical environmental barriers and strategies for improving accessibility and inclusion, while older adults seemed to focus more on social factors (e.g., partnering with organisations so that recreation centres could be a hub for social support; a social atmosphere being important for an inclusive centre culture).

Most participants’ experiences of the recreation centres were positive based on facilities generally meeting their needs and having access to a range of equipment that was mostly in good condition. This is consistent with previous studies which found that these are important factors for supporting recreation centre use among women [46] and adults living with [39, 47] and without [39] disabilities. However, several participants, many of whom had a physical disability or who were aged 65 + years, also identified accessibility barriers of the facilities, such as inadequate disabled parking, inaccessible changing rooms, assistance required for pool access, and poor access routes in and around the centres. These same barriers were identified as common accessibility issues faced by people living with disabilities in a systematic review of studies conducted among adults [48]. Other studies have shown that gender-neutral/family bathrooms, gender-transformed gyms and private or gender-neutral locker rooms are important facilitators for LGBTQIA + persons [32, 34, 49], while parking is important for accessibility for older adults [29].

In our study, participants recommended the provision of more accessible equipment to enhance accessibility and inclusion, paralleling existing research. Previous studies showed beginner/age-appropriate equipment is encouraging for older adults [29] and the provision of aerobic machines is especially important for women [50]. Additionally, having supportive aids for balance, adaptive equipment for gripping, smaller increment weights, removal of physical barriers, wheel-chair friendly surroundings, extra floor space, automatic doors, and family locker rooms have been identified as key for good access for adults living with disabilities [39]. Findings from our study and previous studies suggest differences in the types of facilities that are important for accessibility and inclusion amongst different priority population groups. This understanding is critical for informing best practice universal design principles, which have previously focused on accessibility for persons living with a physical disability in particular, often overlooking other user groups (e.g., LGBTQIA + individuals, older adults, etc.) [51]. While different priority populations had some varying needs, there are certain accessibility and inclusion factors that cater to multiple groups and may provide broad benefit. For example, the provision of sufficient accessible changing rooms (e.g., with shelves and railing) for older adults and individuals living with a disability may also be used as family/gender neutral changing facilities among persons identifying as LGBTQIA+. Ensuring that recreation centres are equipped with adequate and diverse facilities to meet the specific needs of multiple priority populations may be valuable strategies for optimising inclusion and accessibility at recreation centres for all.

Key communication needs identified in this study included having more accessible and inclusive signage (e.g., bigger font on signs for those with vision impairment, multilingual signage) and information provision. This parallels previous research, which has shown that recreation centre use can be facilitated by providing information in other languages [52, 53], distributing information about programs and services targeting specific cohorts [16], and having marketing materials and signage that reflect priority populations (e.g., informative advertisement of inclusion-centred spaces with images of LGBTQIA + people) [32, 39, 49]. Further, a systematic review indicated that signage/information for accessible routes in and around centres and alternative means of accessible information (e.g., braille, large print or audio brochures, tactile cues about location) are important for ensuring that fitness centres are accessible for people living with disabilities [27]. Our findings, coupled with the literature, underscore the importance of ensuring that all communications represent and cater to the needs of various priority populations and that alternative means of communications are available to create inclusive and accessible recreation centres.

Alongside the importance of an appropriate physical environment, the social environment and interpersonal interactions also play a key role in inclusion. Participants cited a social environment and having access to a variety of classes meeting their needs and with good instructors as key factors for fostering positive recreation centre experiences. Similar findings highlighting the appeal of companionship and socialising, in addition to numerous class options, have been observed in previous studies among persons living with disabilities [39, 54], CALD women [52], and older adults [16, 29]. Importantly, social opportunities and a variety of programs may be more important than the functionality of facilities for certain groups (e.g., persons with additional needs and older adults) [47]. When discussing ways for making programs more inclusive and accessible, some participants suggested targeted programs (e.g., graded classes, women’s only programs). This is consistent with previous studies which found that positive experiences at recreation centres can be cultivated by providing specialised classes for persons living with disabilities [39], introductory and graded classes for older adults [16], culturally-familiar programs instructed in native languages for CALD persons [53], women’s only classes [52], and trans-specific and gender non-conforming classes for LGBTQIA + individuals [32]. Several participants, especially those living with a disability, identified instructor training around adaption of programs to accommodate various levels and abilities as a means for improving accessibility and inclusion. This is congruent with previous evidence, which indicated that the lack of adaptive programming was a barrier for adults living with disabilities [27] and that specially trained staff who adapt programs and specific exercises for all fitness levels, hiring staff who are equipped with these adaptive skills, staff training on how to train an individual with a disability, and instructors being responsive to instructions from individuals about what they consider to be the best way to assist them, were facilitators to recreation centre use among this priority population [39, 54]. The provision of targeted classes and specific instructor training may be a promising means for maximising the accessibility and inclusion of recreation centre services, alongside ensuring a social environment that is welcoming to all. Consultation approaches could be considered in future studies to develop recreation services that adequately cater to the specific preferences and requirements of various population groups [55, 56], especially given the differences in needs observed among different priority populations in this study. Co-design is a promising user-centred methodology that could be used for consultation. It incorporates the values, preferences and ideas of the end-users as they engage in continuous reflection and iteration to develop interventions and services while collaborating with researchers and other stakeholders [57]; this approach may enhance community health by meeting community public health needs [58].

Consistent with previous studies among persons living with disabilities [39, 47, 59] and older adults [16, 29], many participants (especially older adults) perceived the social atmosphere and seeing familiar faces as encouraging for centre use as it made for a motivating, inviting culture and was related to feelings of being included. Acknowledgement and engagement from staff were central to perception of inclusion. Our findings and those of other studies among persons living with disabilities [47, 48, 54, 60] and LGBTQIA + persons [32, 49] underscore the importance of staff training on inclusion and accessibility, the need for diverse representation among staff, and the critical role that staff/management can play in combating stigma and cultivating a welcoming environment by applying this knowledge throughout the organisation (e.g., antidiscrimination policies, changing centre norms and values). Future studies may benefit from balancing the needs of various priority groups to ensure both equity and equality. Ensuring that staff are well trained to engage with various priority populations, hiring diverse staff, and fostering a social and welcoming atmosphere for all users may be key strategies for enhancing inclusion and accessibility at recreation centres.

Partnering with community groups was discussed as an opportunity for establishing partnerships with other organisations to improve accessibility and inclusion, aligning with findings from a systematic review among people living with disabilities [61]. The systematic review emphasised the importance of strong relationships between health and recreation sectors and the benefits of partnering with community exercise and sport programs to pool knowledge and resources (e.g., specialised equipment loans and programs) and foster inclusion [61]. Additionally, recreation centre managers and their staff, community groups, organisations that service different priority populations (e.g., persons living with disabilities), city planners, policymakers, and priority populations themselves have all been recognised as important agents of change for addressing barriers to recreation-based exercise participation [59]. Establishing partnerships across sectors and with various stakeholders may be a critical strategy for providing greater opportunities for enhancing the accessibility, inclusion, and service delivery of recreation centres for priority populations.

In summary, we propose several recommendations for enhancing the accessibility and inclusion of recreation centres for priority populations based on our findings, taken in combination with the existing literature. Where possible, accessibility barriers to physical facilities should be addressed, particularly insufficient disabled parking, need for a pool hoist, inaccessible routes in and around centres, and inaccessible changing rooms (e.g., for disabled persons and for LGBTQIA + persons). Although different priority populations have varying needs, the provision of more accessible facilities that meet the needs of multiple groups should be prioritied (e.g., the provision of accessible gender-neutral changing rooms with shelves and railing to cater to persons living with disabilities, older adults, and people identifying as LGBTQIA+). To make communications more inclusive and accessible, it should be provided in multiple languages (e.g., marketing for classes, signage); signage should include enlarged font/text size that is clear and located in visible areas with tactile cues; and centre information (e.g., advertisements for programs) should reflect all types of users in imagery and language, including all priority population groups. In addition to considering the physical environment of recreation centres, efforts should be made to ensure that the social environment is accessible and inclusive by offering a variety of programs and targeted classes for different priority populations (e.g., culturally familiar classes taught in native languages, gender non-conforming classes, graded classes), providing adequate opportunities for social connection, and employing instructors with specialised training and skills in adaptive programming for various sub-groups. Fostering an inviting, motivating culture and welcoming environment for all users is also recommended with a particular focus on having staff trained on inclusion and accessibility, staff members reflecting various priority groups, and having norms and values where inclusion and accessibility are at the core. Lastly, the establishment of partnerships is recommended as it can lead to pooling of resources and knowledge and involve other key agents of change beyond the recreation centre (e.g., city planners, policymakers, community organisations, priority population members), which in turn can make recreation centres more accessible and inclusive for priority populations. Such findings may have applicability to other types of recreational facilities (e.g., sports facilities, physical education infrastructure, commercial gyms, etc.) and various stakeholders (e.g., funders, policymakers, recreation management, urban planners, architects, etc.) and should be further explored in future research.

### Strengths and limitations

This is the first study, to our knowledge, to explore experiences of recreation centres among multiple priority populations alongside the novel investigation of recommendations from these priority groups for enhancing accessibility and inclusion at recreation centres. Another strength is that older adults and persons living with a disability or additional needs were well represented in our sample. Focusing on multiple population groups allows direct comparison and contrasting of experiences and needs. Additionally, this study adds to the literature by evaluating several aspects of recreation centres, which is critical for enhancing knowledge about the progress and success of recreation service delivery, facilities, and programs. A further strength was the collaboration between academia, the local government, and recreation service providers, as such partnerships have been shown to support communities’ public health needs and have been referred to as a “catalyst to improving community health” [58].

There are also limitations to consider. While an equal number of participants in each priority population was sought, this was not achieved with certain priority populations not being represented (e.g., Aboriginal and/or Torres Strait lslanders) or being under-represented (e.g., LGBTQIA+) and others over-represented (e.g., half of the sample was aged 65 + years). We therefore cannot draw meaningful conclusions regarding the under-represented groups of LGBTQIA + persons and people with a CALD background and those who were not represented (Aboriginal and/or Torres Strait Islanders). Recruitment occurred through organisations via the recreation service provider, with no involvement by researchers. This is an appropriate methodology for recruiting priority population groups but precludes control by researchers over the achieved sample. Further research is needed among under-represented priority groups (e.g., persons identifying as LGBTQIA+, people with a CALD background) and priority populations not represented in this study, including First Nations groups who were sought in our study, and persons living with an intellectual disability, who did not form part of the target sample in this study. Despite this, the study still recruited a range of priority populations and had an appropriate sample size for the study design. Many participants represented more than one priority population, posing difficulties with teasing apart the key differences in themes unique to the various priority population groups. However, these are real life circumstances, where most people identify across multiple populations, and although this poses challenges from a research standpoint, each unique perspective is important and valid. It also highlights the importance of recreation centres catering to the needs of all population groups to ensure inclusivity for all. The sample was limited to participants who utilised at least one of three recreation centres in a single LGA in Melbourne, Australia, potentially limiting the generalisability of findings to recreation centres more broadly. However, many of the themes discussed were not specific to a single centre and have been raised in previous literature, suggesting the results have broader relevance. As our study only included recreation centres run by a LGA, it is unclear whether our findings are fully applicable to private gyms and commercial fitness centres, which is an area for future research. The sample comprised users of the recreation centres so did not capture the views of people who were previous users or non-users of such facilities. As this study explored barriers to participation and strategies for enhancing accessibility and inclusion, the lack of priority populations who do not access recreation centres is a key limitation and is an important area for future research. Lastly, although the recreation centres’ facilities and programs offered were primarily indoors, we cannot rule out whether seasonality impacted the findings as the study was conducted during the spring and summer seasons in Australia.

## Conclusion

The present study provided in-depth insights regarding experiences of recreation centres from a broad range of priority populations, as well as their perceptions of strategies for how accessibility and inclusion at these settings can be enhanced, which is paramount for promoting physical activity and ultimately, health for all. While many positive experiences were shared, there were also suggestions for enhancing accessibility and inclusion. There were nuances in the nature of the accessibility barriers and types of suggestions for greater accessibility and inclusion, highlighting the need to consider both the commonalities and the unique needs of different priority populations. A welcoming culture with a variety of programs and facilities and partnerships with community organisations catered to the specific needs of various priority populations are important for fostering accessible and inclusive recreation centres. Our findings can inform the future planning, provision, and enhancement of programs, facilities, and services in recreation centres to reflect the voices and needs of priority populations and reduce inequalities in physical activity opportunities and use of these settings, which in turn can increase physical activity levels and improve population health.

### Electronic supplementary material

Below is the link to the electronic supplementary material.

Supplementary Material 1: Interview Guide. The interview guide includes questions and prompts pertaining to the following pillars of the adapted 7 Pillars of Inclusion model: access and choice (focused on facilities); access and choice (focused on programs); communications; attitude; and partnerships/opportunities

## Data Availability

The data generated and/or analysed during the current study are not publicly available due to confidentiality of the recreation centres and service providers in this study but are available from the corresponding author on reasonable request.

## Abbreviations

LGA: Local Government Area
CALD: Culturally and Linguistically Diverse
SEP: Socio-Economic Position
