# Supplementary material for: Priority populations’ experiences of the accessibility and inclusion of recreation centres: a qualitative study
Source: BMC Public Health. 2024 Jan 17;24:205. doi: 10.1186/s12889-023-17595-3 (PMC10792860; doi:10.1186/s12889-023-17595-3)
Supplement: Supplementary file 1 — Supplementary Material 1: Interview Guide. The interview guide includes questions and prompts pertaining to the following pillars of the adapted 7 Pillars of Inclusion model: access and choice (focused on facilities); access and choice (focused on programs); communications; attitude; and partnerships/opportunities [file 12889_2023_17595_MOESM1_ESM.docx]

Interview Guide

- **PILLAR: Access/Choice**
  - **Facilities (Provide a segue to facilities –“ I will now ask questions concerning the recreation centre(s)’ facilities.” – can tailor these questions to the specific facilities members mention using).**
- What facilities do you use at [state name of recreation centre(s) mentioned]?
  - *After participant has provided responses, then can prompt further - What I mean by facilities are the amenities, parking, transport, building access, toilets, changing rooms, reception, fitness areas, equipment, clear floor space, etc.*
- What has been your experience when using facilities at [state name of recreation centre(s) mentioned]?
- How accessible and inclusive do you find the facilities at [state name of recreation centre(s) mentioned]?
  - *Prompt for participant to expand upon inclusion or accessibility if response only focused on one of the two concepts*; *prompt to get participant to elaborate if answers are brief; prompt for ‘anything else’ until exhausted*.
- Are there any accessibility barriers that you experience when using the facilities?
- What types of facilities or equipment would improve accessibility?
- Are there any inclusion barriers that you experience when using the facilities?
- What types of facilities or equipment would make [state name of recreation centre(s) mentioned] more inclusive?
- How would you suggest changing the facilities to make them more accessible and inclusive?
  - *Prompt – “What kinds of things would you like to see more of or have made available to you?”*
  - *Prompt for participant to expand upon inclusion or accessibility if response only focused on one of the two concepts*.
- What could be changed to improve your experience as a [state name of recreation centre(s) mentioned] user?
- **Programs (*Provide a segue – “I am now going to ask questions related to programs”.*)**
  - What kinds of programs do you do at the [state name of recreation centre(s) mentioned]?

(*Skip follow up questions below if participant does not participate in programs and only uses facilities.*)

- - How easy is it to attend and participate in these programs?
  - Do you feel like the program(s) suits your needs?
  - Are there enough programs that suit you?
  - What types of programs would you like to have available?
  - Do you feel the staff adapt programs to your needs?
  - Are there any barriers you have faced when participating in programs at [state name of recreation centre(s) mentioned]?
  - How would you suggest changing the programs to make them more accessible and inclusive?
    - *Prompt for participant to expand upon inclusion or accessibility if response only focused on one of the two concepts*.
- **PILLAR: Communications (*Provide segue to communications – “I am now going to ask questions about communications.”*)**
  - What are your thoughts on the accessibility and inclusion of communications provided by (state name of recreation centre(s) mentioned)?
    - *Prompt: Communications can include general information provided at the facility, marketing of the programs, communication with the staff, the information and/or user-friendliness of the website, for example.*
  - What do you like?
  - What do you dislike?
  - How would you suggest changing communications [*or could mention a specific thing a participant brings up*] to make them more inclusive and accessible?
  - How often do you want communications and is there a type you prefer?
  - How accessible and inclusive is the [recreation centre(s)’ names] website for you?
  - How would you suggest changing the [recreation centre(s)’ names] website to make it more inclusive and accessible?
- **PILLAR Attitude/Culture (*Provide segue to attitude – “I am now going to ask questions about how you feel as a user and the culture.”)***
- What are your thoughts on the culture of [recreation centre(s)’ names]?
  - Prompt: *What I mean by culture is the collection of values, expectations, and practices that guide and inform the actions of* [recreation centre(s)’ names] *staff and members.*
- How could the culture be changed to make it more inclusive and accessible?
- What are your views on the accessibility and inclusion of the environment at [recreation centre(s)’ names]? This can include facilities, programs, spaces, etc.
  - *Prompt asking how the staff make the participant feel.*
- How included do you feel as a user of [recreation centre(s)’ names]?
  - *Prompt - what could be changed or improved to make you feel more included?*
- What is important for making you feel included as a [recreation centre(s)’ names]?
- What makes it comfortable to be in a [recreation centre(s)’ names]?
- How can your experience be improved to make you feel more comfortable?
- **PILLAR Partnerships/Opportunities (*Provide segue to opportunities– “I am now going to ask questions about further opportunities.”*)**
  - What are other opportunities that could be made available to improve your experience with [recreation centre(s)’ names]?
  - Are there gaps in the service delivery at [recreation centre(s)’ names]?
- Are there certain partnerships that [recreation centre(s)’ names] could have with community organisations to improve accessibility and inclusion?
- Wrap Up:
  - Do you have any final comments or ideas that you would like to express regarding [recreation centre(s)’ names] in general or accessibility and inclusion?

“That concludes the interview. I am now going to stop recording.”

*[Stop recording.]*

“Thank you so much for your time.”

**Interview Complete**
